# Editorial: Annotation and curation of uncharacterized proteins: systems biology approaches

**DOI:** 10.3389/fgene.2015.00224

**Published:** 2015-06-30

**Authors:** Prashanth Suravajhala, Alfredo Benso, Jayaraman K. Valadi

**Affiliations:** ^1^Bioclues.orgHyderabad, India; ^2^Bioinformatics.orgHudson, MA, USA; ^3^Faculty of Health and Medical Sciences, University of CopenhagenFrederiksberg, Denmark; ^4^Politecnico di TurinoTurin, Italy; ^5^Department of Informatics Center, Shiv Nadar UniversityNoida, India

**Keywords:** known unknowns, uncharacterized proteins, regulatory regions, noncoding RNAs, functional genomics, systems biology, annotation

Genomes encode thousands of sequences that play significant roles in diverse biological processes. Understanding the biological functions of these sequences is a challenging, yet promising task. Besides a large set of well-known genes, genomes also contain un-annotated regions whose functions are not known, aptly called “known unknowns” (KU) (Logan, 2009). Research on characterizing the KUs has not only been used to analyze the regulatory effects of genome variation, but also to improve next generation sequencing algorithms. In categorizing functional moieties from hypothetical proteins (HPs), functional genomics has opened new opportunities in identifying the cause for many diseases. For example, the machine learning based approach would allow us to predict sequences for larger sets of the KUs. Although systems biology approaches are applied in classifying the probable functions of these KU products, a few challenges remain.

This research topic gives a synopsis of the current state-of-the art methods to classify and functionally annotate uncharacterized proteins. Ijaq et al. (2015) provide one such framework. The authors discuss the need for functional characterization of HPs with next generation sequencing methods to accelerate multiple areas of genomics, and suggest the use of mass spectrometry as a promising analytical technique in validating protein characterization methods. Discovery and classification of HPs is covered in two papers. Barnkob et al. (2014) present a project designed to collect the necessary data in characterizing the expression of all membrane proteins within the scheme on hematopoietic cells. Another work by Micale et al. (2014) deliberates a way to functionally annotate uncharacterized proteins based on local sequence similarities.

To show how the annotation of HPs may be useful, Ravooru et al. (2014) demonstrate how to annotate uncharacterized proteins with the help of metabolic pathways involved in a known disease. There are two papers that focus on practices to optimize the annotation process. In the first one, Mazandu and Mulder (2014) discuss the problem of comparing genomes annotated using Gene Ontology (GO) terms by proposing a genome-scale approach for integrating annotations from different pipelines using semantic similarity measures. In the second paper, Anton et al. (2014) push for the scientific community to accelerate the rate of gene function validation as a necessary paradigm shift in assigning gene function from the gush of new genome sequences.

Keeping in view the need for rapid identification and characterization of un-annotated proteins, we argue that noncoding RNAs (ncRNAs) may play a large role in understanding the genomic repertoire encoding the un-annotated regions of the genome. We have earlier proposed a six-point classification scoring schema for annotating HPs (Suravajhala and Sundararajan, 2012) and further, the work was projected on the lines of predicting functions using similactors, which are similar proteins and yet not interacting (Benso et al., 2013). We may reason that such approaches may be applicable for these protein interactions. This system-wide omics' approach, we believe would considerably improve the translation of bioinformatics data generated into wet-lab experiments for predicting better drug targets, which in turn may serve as prognostic and diagnostic markers for various diseases (Prensner and Chinnaiyan, 2011). Creating mechanistic archetypes of all uncharacterized regions associated with coding and noncoding genomic repertoire could bring hope for characterizing the uncharacterized.

## Conflict of interest statement

The authors declare that the research was conducted in the absence of any commercial or financial relationships that could be construed as a potential conflict of interest.
